# Supplementary material for: Enhancing food security sustainability through digital information extension services in rural Uganda: Maize postharvest evidence-based strategies
Source: PLoS One. 2025 Nov 6;20(11):e0336105. doi: 10.1371/journal.pone.0336105 (PMC12591458; doi:10.1371/journal.pone.0336105)
Supplement: S1 Appendix — (DOCX) [file pone.0336105.s001.docx]

**S.1 Appendix 1**

**SURVEY QUESTIONNAIRE FOR RURAL SMALLHOLDER MAIZE FARMERS**

**Section A: Background Information**

1. **Gender**: Female Male
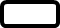

2. **Literacy levels**

I can read and write I cannot read and write

1. **Age**

18 - 20
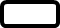


21 – 40

41 – 60

61 – 80
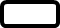


81 and above

1. **Am a member of a financial Group or SACCO (Savings and Credit Cooperative Organization).**
2. YES
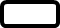
 NO
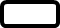

3. **Name of the District and sub counties [tick as appropriate]**

| **Iganga** |  | **Adjumani** |  | **Mubende** |  | **Kabarole** |  |
| --- | --- | --- | --- | --- | --- | --- | --- |
| 1.Bulamagi |  | 1.Adropi |  | 1.Kitenga |  | 1.Kasenda |  |
| 2.Nabitende |  | 2.Pachara |  | 2.Kalonga |  | 2.Ruteete |  |
| 3.Nakalama |  | 3.Ciforo |  | 3.Kayebe |  | 3.Mugusu |  |
| 4.Nakigo |  | 4.Ukusijoni |  | 4.Madudu |  | 4.Busoro |  |
| 5.Nambale |  | 5.Pakele |  | 5.Kiyuni |  | 5.Kabende |  |
| 6.Namungalwe |  | 6.Dzaipi |  | 6.Butoloogo |  | 6.Kicwamba |  |
| 7. Nawandala |  | 7.Arinyapi |  | 7.Kiruuma |  | 7.Karangura |  |
| 8.Nawaningi |  | 8.Ofua |  | 8.Kasambya |  |  |  |
|  |  | 9.Itirikwa |  | 9.Kigando |  |  |  |
|  |  |  |  | 10.Nabingoola |  |  |  |
|  |  |  |  | 11.Lubimbiri |  |  |  |
|  |  |  |  | 12.Kibalinga |  |  |  |
|  |  |  |  | 13.Bagezza |  |  |  |

**Section B: Current Status of Maize Postharvest handling**

1. **What was the total acreage of land under maize cultivation for the last season**

Less than one (1) acre
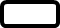
 One (1) to five (5) acre
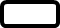


1. **What was the estimated quantity of maize you harvested last season? (One bag = 100kgs)**

Less than 10 bags
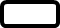


10 bags to 50 bags
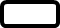


51 bags and above
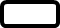


1. **What was the estimated quantity of maize you lost after harvest due to postharvest challenges? (One bag = 100kgs)**

Less than 10 bags
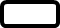


10 bags to 50 bags
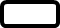


51 bags and above
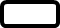


No maize postharvest loss experienced
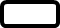


**Section C: Access and use of evidence based information influence on maize postharvest handling.**

***Please tick the most appropriate response to the statement for question 9 to 16***

1. Before storage of maize, I access evidence-based information to influence my decisions and practices. 9a. Strongly agree 9b. Agree
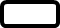
 9c. Not sure
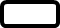


9e. Disagree
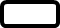
. 9f. Strongly Disagree
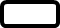


1. Before storage of maize, I use evidence-based information to influence my decisions and practices. 10a. Strongly agree
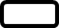
 10b. Agree
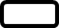
 10c. Not sure
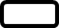


10e. Disagree
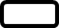
 . 10f. Strongly Disagree
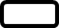


1. Before drying of maize I access evidence-based information to influence my decisions and practices. 11a. Strongly agree
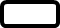
 11b. Agree
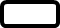
 11c. Not sure
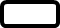
 11d. Disagree
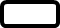
 11e. Strongly Disagree
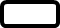

2. Before drying of maize, I use evidence-based information to influence my decisions and practices. 12a. Strongly agree
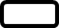
 12b. Agree
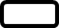
 12c. Not sure
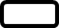
 12d. Disagree
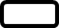
 12e. Strongly Disagree
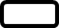

3. Before selling of maize, I access value addition evidence-based information to influence my decisions and practices. 13a. Strongly agree
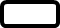
 13b. Agree
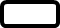
 13c. Not sure
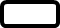


13d. Disagree
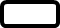
 13e. Strongly Disagree
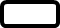


1. Before selling of maize, I use value addition evidence-based information to influence my decisions and practices. 11a. Strongly agree
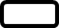
 14b. Agree
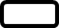
 14c. Not sure
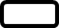
 14d. Disagree
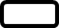
 14e. Strongly Disagree
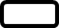

2. Before maize postharvest handling, I access financial information to influence my financial decisions and practices 15a. Strongly agree
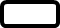
 15b. Agree
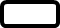
 15c. Not sure
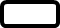
 15d. Disagree
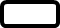
 15e. Strongly Disagree
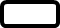

3. Before maize postharvest handling, I use financial information to influence my financial decisions and practices 16a. Strongly agree
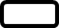
 16b. Agree
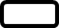
 16c. Not sure
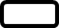
 16d. Disagree
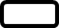
 16e. Strongly Disagree
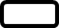

4. Before maize postharvest handling, I participate in continuous training to gain knowledge on how to handle maize effectively. 13a. strongly agree
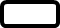
 13b. Agree
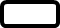
 13c. Not sure
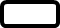
 13d. Disagree
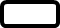
 13e. Strongly Disagree
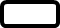

5. What Evidence-based Information Practices are you aware of? [Tick as many as applicable]

Asking farmers’ information needs on maize postharvest handling
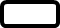


Acquisition of maize postharvest information
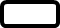


Appraising maize postharvest handling information for credibility and quality
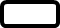


Aggregating maize postharvest handling information for easy access and use
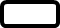


Applying maize postharvest handling information for better decisions and practice
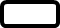


Assess maize post-harvest handling information application for improvement
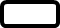


No evidence-based information practices are available
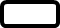


1. Source of evidence-based information practices implemented in Uganda? [Tick as many as applicable]

Village meetings/church meetings
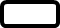


Agricultural Extension Office
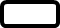


Through Nongovernmental Organizations (NGOs)
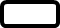


Through personal experience/ learning offline or online means
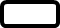


Through District/Village/Sub county Information Officers
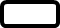


Community Libraries
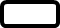


Friends
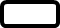


Media (Radio/Tv/Newspapers)
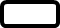


I do not know any source
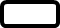


1. Tick the following stakeholders whom you know support you with evidence-based information. [Tick as many as applicable]

National Agricultural Advisory Services (NAADS)
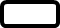


District Extension Officers
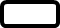


World Food Programme (WFP)
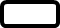


Food and Agricultural Organization (FAO)
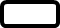


National Agricultural Research Organization (NARO)
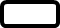


Agriculture Cluster Development Project
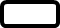


Community Librarians
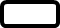


1. Which are the specific maize postharvest handling evidence-based information practice needs for rural smallholder farmers in Uganda? (Tick as many as applicable)

Drying
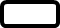


Storage
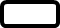


Value addition
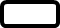


Credit support
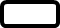


Continuous training
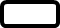


Not mention
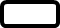
 (specify)

1. Do the current maize postharvest evidence-based information practices influence your daily decisions and practices?

YES
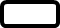
 NO
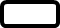


1. What are the difficulties you experience with the current evidence-based information practices in Uganda? [ Tick as appropriate according to the scale: 1- Strongly agree, 2- agree, 3- not sure 4- disagree, 5- strongly disagree

| **No** | **Difficulties** | **1** | **2** | **3** | **4** | **5** |
| --- | --- | --- | --- | --- | --- | --- |
|  | Evidence-based information is not adequate enough to empower small farmers to overcome post-harvesting difficulties |  |  |  |  |  |
|  | Farmers walk long distances to access evidence-based information |  |  |  |  |  |
|  | Farmers are not aware of hermetic or any other evidenced storage facilities |  |  |  |  |  |
|  | Farmers do not know possible technologies for value addition methods |  |  |  |  |  |
|  | Farmers are not aware of credit/financial facilities available to support postharvest handling |  |  |  |  |  |
|  | Training provided are ad hoc in nature so farmers do not benefit |  |  |  |  |  |
|  | Lack of evidence-based information practice framework causes all the problems mentioned above |  |  |  |  |  |
|  | No known source of maize postharvest handling evidenced information |  |  |  |  |  |
|  | The difficulties we experience is not mentioned here. It is……………….. |  |  |  |  |  |
|  | No difficulties experienced |  |  |  |  |  |

**D: Proposed recommendations by Rural Maize Smallholder Farmers in Uganda**

1. What would you like to be included in the evidence-based information practice framework for maize postharvest handling by smallholder farmers in Uganda? Tick as appropriate according to the scale: 1- Strongly agree, 2- agree, 3- not sure 4- disagree, 5- strongly disagree

| **No** | **Recommendations** | **1** | **2** | **3** | **4** | **5** |
| --- | --- | --- | --- | --- | --- | --- |
| 1. | A mechanism for involving smallholder farmers to share their evidence-based information needs in terms of drying, storage, value addition, credit access, and training |  |  |  |  |  |
| 2. | A mechanism that can enable smallholders to access adequate and convenient evidence-based information |  |  |  |  |  |
| 3. | A mechanism that can ensure the evidence-based information practice is (Ask, Acquire, Appraise, Aggregate, Apply and Assess) implemented |  |  |  |  |  |
| 4. | A mechanism that can ensure continuous training is done for smallholder farmers |  |  |  |  |  |
| 5. | A mechanism to ensure smallholder farmers access information on financial/credit support |  |  |  |  |  |
| 6. | All of the above-mentioned mechanisms |  |  |  |  |  |
| 7. | Not mentioned above: It is……………………………………………………………. |  |  |  |  |  |

1. Why do you say the mechanisms mentioned in 18 above should be included in the framework?

**Tick as appropriate according to the scale: 1- Strongly agree, 2- agree, 3- not sure 4- disagree, 5- strongly disagree**

| **No** | **Reasons** | **1** | **2** | **3** | **4** | **5** |
| --- | --- | --- | --- | --- | --- | --- |
| 1. | Because a framework will ensure all the necessary inputs into implementation of an evidence-based information practice are included |  |  |  |  |  |
| 2. | The framework will ensure smallholder farmers are empowered with the knowledge on specific evidence-based information needs in terms of drying, storage, value addition, credit access, and training. |  |  |  |  |  |
| 3. | The framework will ensure smallholders are empowered to access adequate and convenient evidence-based information. |  |  |  |  |  |
| 4. | The framework will ensure the evidence-based information practices (Ask, Acquire, Appraise, Aggregate, Apply and Assess) for maize postharvest by smallholder farmers are implemented. |  |  |  |  |  |
| 5. | A framework will ensure continuous training is done for smallholder farmers. |  |  |  |  |  |

**THANK YOU**
