# Supplementary material for: Enhancing food security sustainability through digital information extension services in rural Uganda: Maize postharvest evidence-based strategies
Source: PLoS One. 2025 Nov 6;20(11):e0336105. doi: 10.1371/journal.pone.0336105 (PMC12591458; doi:10.1371/journal.pone.0336105)
Supplement: Attachment — Submitted filename: Response to reviewers.docx [file pone.0336105.s003.docx]

**Jackline Estomihi Mayende Kiwelu,**
Aga Khan University

Urambo Street, Upanga
P. O. Box 125

Dar es Salaam

Tanzania

06/10/2025.

**Editor-in-Chief**
*Plos One Journal*.

**REF: Response to the Academic editor and Reviewers on Manuscript PONE-D-25-39333 entitled: Enhancing food security sustainability through digital information extension services in rural Uganda: maize postharvest evidence-based strategies**

We sincerely appreciate you and the reviewers for your time and effort in improving our manuscript. The constructive feedback has greatly improved the readability of our paper.

We have carefully addressed all comments and revised the manuscript accordingly. Below, we provide a detailed, point-by-point response to each editor’s and reviewer’s comments. For ease of reference, reviewers’ comments are presented in bold, followed by our responses in regular text. All changes have been highlighted in the revised manuscript through tracked changes method.

We have read the PLOS open data access policy and complied by providing the link to the data in the drive one folder. My institution does not yet have an open data repository. I am willing to provide this folder in case Plos One has an open data repository to upload it, and then we may use the link for the public. Any further details can be sought through the corresponding author at [jackline.kiwelu@aku.edu](mailto:jackline.kiwelu@aku.edu)

Your sincerely

**Jackline Estomihi Mayende Kiwelu**

**Response to Editors and Reviewers:**

Editors

1. Please ensure that your manuscript meets PLOS ONE's style requirements, including those for file naming. The PLOS ONE style templates can be found at <https://journals.plos.org/plosone/s/file?id=wjVg/PLOSOne_formatting_sample_main_body.pdf>

<https://journals.plos.org/plosone/s/file?id=ba62/PLOSOne_formatting_sample_title_authors_affiliations.pdf>

**Response to the editor:** The researchers have used the PLOS ONE Style as required.

2. When completing the data availability statement of the submission form, you indicated that you will make your data available on acceptance. We strongly recommend all authors decide on a data sharing plan before acceptance, as the process can be lengthy and hold up publication timelines. Please note that, though access restrictions are acceptable now, your entire data will need to be made freely accessible if your manuscript is accepted for publication. This policy applies to all data except where public deposition would breach compliance with the protocol approved by your research ethics board. If you are unable to adhere to our open data policy, please kindly revise your statement to explain your reasoning and we will seek the editor's input on an exemption. Please be assured that, once you have provided your new statement, the assessment of your exemption will not hold up the peer review process.

**Response to the editor and reviewer one:** The data is available in the drive one folder [at https://aku4-my.sharepoint.com/:f:/g/personal/jackline_kiwelu_aku_edu/EprbLR-CGk1Ki_opjEjP8O4Bq3BBo8lbVbnuI_0A8F8HQQ?e=6sQUaN](https://aku4-my.sharepoint.com/:f:/g/personal/jackline_kiwelu_aku_edu/EprbLR-CGk1Ki_opjEjP8O4Bq3BBo8lbVbnuI_0A8F8HQQ?e=6sQUaN)

2. Your ethics statement should only appear in the Methods section of your manuscript. If your ethics statement is written in any section besides the Methods, please move it to the Methods section and delete it from any other section. Please ensure that your ethics statement is included in your manuscript, as the ethics statement entered into the online submission form will not be published alongside your manuscript.

**Response to the editor**: It is available in the page Methods section on page 17 as required

4. Please include a copy of Table 6 which you refer to in your text on page 29.

**Response to the editor**: It was a mistake in renaming. It has been corrected.

**Reviewer One**

i. **Reviewer comment: The analysis was not vigourous**

**Response:**

The statistical analysis was performed appropriately and rigorously following standard procedures. Data were analyzed using SPSS and Atlas ti for descriptive statistics were used to summarize participant characteristics, and inferential tests such as Chi-square test, were applied to examine associations between variables, in line with the study objectives. Assumptions for each statistical test were verified before analysis. The level of significance was set at p < 0.05. We have clarified this in the Methods and Material section.

ii. We agree that the statement in the introduction on page 6 could appear too general without contextualizing farmers' specific postharvest practices.

**Response to Reviewer one:** To address this, we have revised the section to include examples of how farmers typically harvest, dry, store, and transport maize and how these practices influence mould growth, termite infestation, and aflatoxin contamination. This addition strengthens the link between farmers’ practices and the need for effective digital agricultural extension information services. As seen Page 6

*“With issues such as mould, termites, and aflatoxin contamination continuing to undermine maize quality (11), farmers often face persistent postharvest losses and health risks. These challenges are closely linked to traditional practices such as harvesting maize before full maturity, drying cobs on bare ground, storing in poorly ventilated structures, and transporting in non-airtight bags. Such practices increase exposure to moisture, pests, and fungal infection. Providing farmers with effective digital agricultural extension information services is therefore critical to enhance food security across four dimensions (Amin & Mehrez, 19). These services can offer real-time guidance on appropriate harvesting times, improved drying methods, safe storage technologies, and better transport practices, thereby reducing losses and improving food safety”*

iii. We agree that the Hypothesis Testing section on page 16 contains repetitive phrasing for each EBIP and could be more concise.

**Response to Reviewer one:** To address this, we have revised the section by summarizing the hypotheses collectively, indicating that each of the six 6As was tested under the null assumption of no significant relationship. We have also added a clear statement on the statistical method used for hypothesis testing as see below:

“*In this study, hypotheses were formulated for each of the six 6As, with the null hypothesis stating that no significant relationship existed between each EBIP and the maize postharvest handling. We employed Pearson’s chi-square product-moment correlation to test these hypotheses, which allowed us to examine whether statistically significant associations were present.”*

iv. The questionnaire details were added as suggested by the reviewer and the questionnaire attached as appendix 1.

**Response to Reviewer one**: Quantitative data were gathered using a structured questionnaire. The instrument consisted of both closed and open-ended questions organized into five sections: (a) Background information, (b) current status of maize postharvest handling, (c) access and use of EBI, (d) recommendations. The questionnaire was administered only to rural maize smallholder farmers. The complete questionnaire is included as Appendix 1 as seen in page 52

v. Page 19, ATLAS.ti 24: The authors indicated they used ATLAS.ti 24. What is ATLAS.ti 24?

**Response to Reviewer one:**
 We have briefly described the software and its role in our analysis as :

Qualitative data were analyzed using **ATLAS.ti 24**, a computer-assisted qualitative data analysis software that supports systematic coding, organization, and retrieval of textual and visual data.

vi. Page 21, FGD: This acronym needs to be spelled out, as the reader does not know what “FSD” is.

**Response to reviewer one**: We have improved to read : Focus Group Discussion (FGD)

vii. Page 22, Table 2 and Page 26, Table 3: What is “SACCO”? This acronym needs to be spelled out in a footnote to Tables 2 and 3.

**Response to reviewer one**: It has been done as suggested Savings and Credit Cooperative Organization (SACCO).

viii. Response to reviewer one: The description of the 6As (evidence-based information practices) in page 10 the paragraph that starts with leveraging, provides the context. However, the researchers have added some more context to the practices themselves.

ix. Abstract and the reporting in Table 4 lacked sufficient context and detail.

**Response to reviewer one:**

To address this, we have revised the manuscript to provide (i) a clear explanation of what the 6As represent in this study, (ii) how each EBIP was operationalized and quantified, and (iii) the meaning of the “Number” is responses ‘YES ‘column in Table 4.

As seen below:

the “Number” column refers to the count of maize farmers who confirmed they were aware that rural extension officers practiced the corresponding EBIP. We have also clarified that “*the information was obtained from farmers’ responses to structured questions in the questionnaire, where they were asked whether they observed or received each of the listed EBIPs from extension officers. as seen in the questionnaire question 19.*

x. **Response to reviewer one** :

Figure 3: has been improved. Figure 4 has been revised and improved.

xi. Response to reviewer one: Table 4 and Table five have been combined as suggested by the reviewer one

Reviewer two

1. Summarise the Introduction.

**Response to reviewer two:** The researchers have summarized it from 5 pages to 4.
